# Supplementary material for: Patterns of Intron Gain and Loss in Fungi
Source: PLoS Biol. 2004 Nov 30;2(12):e422. doi: 10.1371/journal.pbio.0020422 (PMC532390; doi:10.1371/journal.pbio.0020422)
Supplement: Table S1 — Also available at http://genes.mit.edu/NielsenEtAl/. (4.3 MB ZIP). [file pbio.0020422.st001.zip › NielsenEtAl/html/181.html]

AN5734.1.NCU00988.1.MG04225.1.FG06235.1


```
 CLUSTAL W (1.82) Multiple Sequence Alignments - Introns Inserted


Sequence 1: NCU00988.1	537 aa
Sequence 2: MG04225.1	536 aa
Sequence 3: AN5734.1	524 aa
Sequence 4: FG06235.1	531 aa
Alignment Length: 542 aa
Number Identitical Residues: 244 aa
Alignment Score (without introns) 12322


MG04225.1 	MGKAS-VKVLQKIVR0NDAMHTDPPEIYGWRVYTLAMS0ACFGAMCFGWDSSVIGGVIVL
NCU00988.1	MGLSIGNRILRKIVK~NEAMAEDPPEIYGWRVYLLACS0ACFGAMSFGWDSSVIGGVIEL
FG06235.1 	MGIAG---VLKKVVR~NDAMKTDPEEIYNWRVFAVVAG~SCFGGMLFGWDTGAIGGVLAM
AN5734.1  	-----MSFLLRKLVH~NEAMRTDPREIYGWRVYALACS0ACFGGMLFGVETGIIGGVLTM
          	        :*:*:*: *:**  ** ***.***: :. . :***.* ** ::. ****: :

MG04225.1 	DAFKA~DYGLSPDSKDYRSQDLAANIVSTLQAGCFLGAIVASPMTDAFGRRRCLLGVAGI
NCU00988.1	EPFKH~DFGFIGNDK--AKANLGANIVSTLQAGCFLGALIASPITDRFGRKWCLIAVSLV
FG06235.1 	KATQE~RFNYTPEAK----VTLDQNIVSTLQAGCFAACFFTSYFTERYGRRWCLIGTGAV
AN5734.1  	DTFKA2KYGLN-ELGDTGRANLSANIVSTLQAGCFFGALAASWVADKYGRRLGLIAASFV
          	.. :   :.   :  .     *  *********** ..: :* .:: :**:  *:... :

MG04225.1 	IMIGVIMQGA--ASGSLGALYAGR~FVAGFGVGAASCINPIYVSENAPRAIRGLLTG~MY
NCU00988.1	VIIGIIMQAA--ASGNLAPMYIGR~FVAGVGVGAASCINPVFVSENAPRSIRGLLTG~LY
FG06235.1 	TTIGVILQAASTAQGSLPVMYIGR~FVAGLGVGAASSLVPLYVSECAPRAIRGGLTS1FY
AN5734.1  	ALVGVIMQVA--ASGHLEAMYIGR2LLCGIGVGFASMVNPLYVSENAPRAIRGGLTG~LY
          	  :*:*:* *  *.* *  :* ** ::.*.*** ** : *::*** ***:*** **. :*

MG04225.1 	QLFIVTGGMIAFW~INYAVNLHHRGKTSYILPLSVQGVPAA~LLFITMWMCKES~PRWLA
NCU00988.1	QLFIVTGGMIAFW~INYSVSLHFKGKSMYIFPLAIQGLPAG~LLCVCMLLCHES~PRWLA
FG06235.1 	QLFIVTGVMLSFW2INYGALLHLSAPTVYALPLALQALPAV2LLIACMLAAPES2PRWCA
AN5734.1  	QLFITMGIMLAFW~INYGSLLHISGAGQYLVPLAMQGLPAI~LLLVGMLLCNES~PRWLA
          	****. * *::** ***.  **  .   * .**::*.:**  **   *  . ** *** *

MG04225.1 	RKDRWEEARAVLSHTRN~LPSDHPYLQEEMAEIADQLEQERRLVGDASWLQLNKEIWRQS
NCU00988.1	RRDRWEECKSVLARIRN~LPPDHPYIVDEFREIQDQLEQERRLQGDATYWDLTRDMWTVA
FG06235.1 	RKDDWERAKSILIGLRQ2LPETHPYVQTELQEMSAQLEAEKRLTGNASASTLWKELVTIP
AN5734.1  	KQDRWEEARATLSRVRN~LPSTHPYVENEFQDIVRQLEFERQLISGSSFVDLLKEMWFIP
          	::* **..:: *   *: **  ***:  *: ::  *** *::* ..::   * :::   .

MG04225.1 	IHRKRAIISIVLMICQQM0TG~TNAINTYAPTIFANLGIKGLDGQLLGTGVYGV0VKVIS
NCU00988.1	GNRKRALISIFLMICQQM~TG~TNAINTYAPTIFKNLGITGTSTSLFSTGIYGI~VKVVS
FG06235.1 	GNRKRAIISVLLMVCQQM~TG1VNAVNYYAPQIFQSLGMTGTTVSLFATGVYGI~IKVLG
AN5734.1  	GNRKRALISIWLMICQQM~TG~TNAINYYAPQIFENLGITGSANGLFATGVYGI~VKVVG
          	 :****:**: **:**** ** .**:* *** ** .**:.*    *:.**:**: :**:.

MG04225.1 	CIAFLLFMADSLGRRRSLLWTSIAQGLVMFYIGIYVRIAPPKDGDPVPPAGYFALVCIFL
NCU00988.1	CVIFLLFLADSLGRRRSLLWTSIAQGLAMFYIGLYVRISPPIDGQPVPPAGYVALVCIFL
FG06235.1 	CATFLIFFADSLGRRRSLLWTSAAQFLAMYIIGIYGRVEPPVAGAGISAFGYVAIVCIYL
AN5734.1  	CACFLVFVADSLGRRRSLLWTSIAQGLAMLYIGLYVRIAPPKEGEPVIPAGYFALVCIFL
          	*  **:*.************** ** *.*  **:* *: **  *  : . **.*:***:*

MG04225.1 	FAA2FFQFGWGPACWILASEIPSARFRSINVSYAAATQWLFNFVVARSVPVMLITIG2GK
NCU00988.1	FAA2FFQFGWGPACWIYASEIPAARLRSLNVSYAAATQWLFNFVVARAVPTMLVTVG~-P
FG06235.1 	WAA~FFQFGWGPCCWILVSEIPTARLRALNVAIAAATQWLFNFIVARTVLTMQKTMG~-P
AN5734.1  	FAA~FFQFGWGPVCWIYVSEIPTTRLRSLNVSFAAATQWLFNFVVARAVPNMLATVG~-A
          	:** ******** *** .****::*:*::**: **********:***:*  *  *:*   

MG04225.1 	TGY~G~TYLLFGSFAFTMFFFVWFFVPETKG~LSLEAMDELFGGS--PSGETLKNTQLSE
NCU00988.1	HGY~G~TYLIFGSFCLSMFVFVWFFVPETKG~ISLEHMDELFGVTDGPAAEKSSVHGGDD
FG06235.1 	AGY0G~MFFMFGTFDLLMGIFVWLFVPETKG~LSLEQMDELFGVADVQGKLDAEGPEGAR
AN5734.1  	NGY~G2AYIIFACFCFSMFVFVWFFVPETKG1LSLEKMDDLFGISHGSTKESADESP-AE
          	 ** *  :::*. * : * .***:******* :*** **:*** :        .      

MG04225.1 	NADETTVVPMPPKQYGLA-
NCU00988.1	VGSEMGKGDQKSKHVEVYV
FG06235.1 	TPSIREEVAHIKSNKS---
AN5734.1  	KQEEIRIEKTA--------
          	  .
```
